# Supplementary material for: Evaluation of sea turtle morbidity and mortality within the Indian Ocean from 12 years of data shows high prevalence of ghost net entanglement
Source: PLoS One. 2023 Aug 9;18(8):e0289167. doi: 10.1371/journal.pone.0289167 (PMC10411791; doi:10.1371/journal.pone.0289167)
Supplement: Attachment — Submitted filename: Himpson_etal_PONE_Maldives_entanglements_review.docx [file pone.0289167.s002.docx]

Himpson et al. PONE

Evaluation of sea turtle morbidity and mortality withi the central Indian Ocean from 12 years of data shows high prevalence of ghost net entanglement

Reviewed for PLOS ONE (Manuscript: PONE-D-22-34145 Deadline 19/01/23)

This sea turtle study presents 12 years of stranding and rehabilitation data from the Maldives to report key threats to sea turtles, identifying entanglement in ghost nets as one of the key anthropogenic causes of injuries. The dataset is considerable and includes 459 turtles. Clear study objectives are presented including variation in cause of injury between species and life stages, estimation of mortality rate and identification of seasonality in strandings. Providing that the review comments below are addressed, the study would advance our understanding of some of the underreported anthropogenic impacts from fishing, as already highlighted by a number of studies in the region (e.g., Stelfox et al) cited in the manuscript.

The findings are clearly presented but the manuscript could be much improved by re-arranging content into subsections with relevant subtitles in Methods and Results. No figures and/or tables were included so it was difficult to interpret results. Additionally, no underlying data were provided (that I could access) so it was difficult to comment on the review question ‘Have the authors made all data underlying the findings in their manuscript fully available?’. To this end, a supplementary table providing summary data would be helpful to support the growing body of literature on this topic in addition to providing a link to the Reefscapers website.

Some specific comments:

Abstract:

State number of atolls and relative location where data were collected

The study is for one small area of the Indian Ocean and I suggest re-phrasing ‘high prevalence of ghost net entanglement in the Indian Ocean’. If a review has shown high prevalence elsewhere in the IO then include the results.

Introduction:

I recommend to commence with a paragraph describing different threats to turtles (and introducing the various anthropogenic threats) before focusing on species and assessment.

L77: define what you are including in the term Central Indian Ocean to help with readers’ understanding as typically this region is split between NWIO and SWIO and NEIO with respect to sea turtle regional management units (RMUs) Wallace et al. 2010. <http://www.doi.org/10.1371/journal.pone.0015465>

L79: insert the citation as numeric.

L84: provide more context about population connectivity in the Western Indian Ocean, for example a number of green turtles have migrated to Maldives from Chagos Archipelago in the south (Hays et al. 2020 <https://doi.org/10.1016/j.cub.2020.05.086>) which provides critical nesting habitat for turtles from around the region (see Mortimer et al. 2020 <http://www.doi.org/10.1017/S0030605319001108>).

Results:

I would suggest to the authors to provide a number of graphs to show key results, for example:

To support the statements about turtles stranded or bycaught turtles in the Maldives : provide a map with indication of numbers of each case category at each location

To support statements about seasonal patterns of admissions : provide a graph comparing number of cases per month of the year

To support statements about entanglement being key cause of injuries or death: graph reporting number of cases for each threat identified

Provide a graphical overview of relative proportion of life stages and species included in the study

Discussion:

This is quite lengthy and could be improved by re-ordering to commence the section with key results that are discussed in context of the literature, followed by several sections with appropriate sub-titles to discuss the various findings. There are some statements that should only be included if supported by citations (e.g. L418 turtle populations in the Central IO are some of the most threatened in the world …)

It is worthwhile considering potential bias in the dataset due to method of entanglement. Relative contributions of ghost nets to entanglement and injury/mortality of turtles is possibly overreported compared to other causes of anthropogenic threat. This is because ghost nets are likely to come ashore as they are usually buoyant and carried in surface waters. Turtles that snag on fishing line caught on the reefs are less likely to be included in the dataset. Consider other anthropogenic factors in your discussion, e.g. those reported by Casale et al. 2010 <https://doi.org/10.1002/aqc.1133>

L294: As suggested for the introduction, a definition of the area (Central Indian Ocean) and a map of the study site would help support this statement.
